# Saccade-Responsive Visual Cortical Neurons Do Not Exhibit Distinct Visual Response Properties

**DOI:** 10.1523/ENEURO.0051-23.2023

**Published:** 2023-09-13

**Authors:** Chase W. King, Peter Ledochowitsch, Michael A. Buice, Saskia E. J. de Vries

**Affiliations:** 1 MindScope Program, Allen Institute, Seattle, Washington 98109; 2 Department of Computer Science, University of Washington, Seattle, Washington 98195-2350; 3 Department of Applied Mathematics, University of Washington, Seattle, Washington 98195-3925; 4 Department of Physiology & Biophysics, University of Washington, Seattle, Washington 98195-7290

**Keywords:** calcium imaging, mouse visual cortex, saccades, transgenic lines

## Abstract

Rapid saccadic eye movements are used by animals to sample different parts of the visual scene. Previous work has investigated neural correlates of these saccades in visual cortical areas such as V1; however, how saccade-responsive neurons are distributed across visual areas, cell types, and cortical layers has remained unknown. Through analyzing 818 1 h experimental sessions from the Allen Brain Observatory, we present a large-scale analysis of saccadic behaviors in head-fixed mice and their neural correlates. We find that saccade-responsive neurons are present across visual cortex, but their distribution varies considerably by transgenically defined cell type, cortical area, and cortical layer. We also find that saccade-responsive neurons do not exhibit distinct visual response properties from the broader neural population, suggesting that the saccadic responses of these neurons are likely not predominantly visually driven. These results provide insight into the roles played by different cell types within a broader, distributed network of sensory and motor interactions.

## Significance Statement

While the existence of saccade-responsive neurons in visual cortex has been previously established, how these responses vary by transgenically defined cell type and whether these neurons differentially process visual stimuli are unknown. In addition to identifying distinct neural cell types more likely to robustly respond to saccades, our results indicate that these saccade-responsive neurons do not exhibit distinct visual responses from their non-saccade-responsive counterparts.

## Introduction

Saccades are rapid eye movements that quickly shift the visual field between two points of fixation. Unlike primates, mice have no fovea, yet numerous studies have previously found that mice make saccades in both freely moving and head-fixed contexts (Sakatani and Isa, 2007; Samonds et al., 2018; Meyer et al., 2020). In the head-fixed context, these saccades are almost exclusively conjugate—both eyes moving in unison in the same direction—and occur preferentially along the horizontal direction (Samonds et al., 2018; Meyer et al., 2020). It has also been previously established that neurons in primary visual cortex (V1) modulate their responses to these saccadic eye movements (Roth et al., 2016; Miura and Scanziani, 2022).

Recent experimental findings in mice implicate the lateral posterior (LP) nucleus, a higher-order thalamic nucleus that is the rodent homolog of the thalamic pulvinar nucleus, as the primary nonvisual source of saccade signals in cortex (Roth et al., 2016; Miura and Scanziani, 2022). The LP is innervated extensively by the superior colliculus (SC; Zhou et al., 2017; Leow et al., 2022), a midbrain structure important for sensorimotor transformation that has been shown to contain a topographic eye movement map capable of evoking eye movements (Gandhi and Katnani, 2011; Wang et al., 2015; Zahler et al., 2021). Cortically, LP projects to layers 1 and 5 in V1, and to layers 1 and 4 in higher visual areas (Herkenham, 1980). Recent electrophysiological work has found that this LP saccade signal encodes saccade direction and contributes to a subset of V1 neurons responding to eye movements in a direction-selective (DS) manner (Miura and Scanziani, 2022).

While it has been well established that saccade-responsive (SR) neurons exist in visual cortex, it remains unknown, however, how these saccade-responsive neurons are organized. In the present study, we seek to address the following: (1) how saccade-responsive neurons are distributed across different visual areas, cortical layers, and genetically defined cell types; and (2) how visual response properties of these saccade-responsive neurons compare to the visual response properties of the broader neural population. Specifically, we examined the saccadic eye movement behaviors across transgenic lines of mice and characterized the saccade response properties of different types of cells across visual areas and cortical layers. We analyzed physiological data from the Allen Brain Observatory Visual Coding dataset (publicly available at https://portal.brain-map.org/ and the Allen SDK Python package), which consists of calcium imaging recordings in head-fixed mice across 6 visual areas, 4 cortical layers, and 14 transgenically defined cell types in the mouse visual cortex (Table 1; Allen Brain Observatory – 2-Photon Visual Coding, 2016; de Vries et al., 2020). We found saccade-responsive neurons distributed across all visual cortical areas, layers, and cell types within this dataset. However, this distribution was not uniform, as we observed comparatively more saccade-responsive neurons in certain classes of inhibitory interneurons and in deeper cortical layers. Furthermore, we found that these saccade-responsive neurons do not have distinct visual response properties, suggesting that saccade responses are not a uniquely visually driven response.

**Table 1:** Transgenic line abbreviations

Abbreviation	Full transgenic line	Number of mice	Number of sessions	Number of neurons	Number of neurons analyzed
Emx1;Ai93	Emx1-IRES-Cre; Camk2a-tTA; Ai93(TITL-GCaMP6f)	17	83	8866	5020
Slc17a7;Ai93	Slc17a7-IRES2-Cre; Camk2a-tTA; Ai93(TITL-GCaMP6f)	24	124	8512	7707
Slc17a7;Ai94	Slc17a7-IRES2-Cre; Camk2a-tTA; Ai94(TITL-GCaMP6s)	1	6	817	817
Cux2;Ai93	Cux2-CreERT2; Camk2a-tTA; Ai93(TITL-GCaMP6f)	32	121	10181	6991
Rorb;Ai93	Rorb-IRES2-Cre; Camk2a-tTA; Ai93(TITL-GCaMP6f)	18	61	4738	3485
Scnn1a;Ai93	Scnn1a-Tg3-Cre; Camk2a-tTA; Ai93(TITL-GCaMP6f)	6	21	1513	838
Nr5a1;Ai93	Nr5a1-Cre; Camk2a-tTA; Ai93(TITL-GCaMP6f)	20	71	2122	1353
Rbp4;Ai93	Rbp4-Cre_KL100; Camk2a-tTA; Ai93(TITL-GCaMP6f)	21	72	1649	1399
Fezf2;Ai148	Fezf2-CreER; Ai148(TIT2L-GC6f-ICL-tTA2)	8	25	1346	1338
Tlx3;Ai148	Tlx3-Cre_PL56; Ai148(TIT2L-GC6f-ICL-tTA2)	3	12	979	979
Ntsr1;Ai148	Ntsr1-Cre_GN220; Ai148(TIT2L-GC6f-ICL-tTA2)	9	33	1180	1180
Sst;Ai148	Sst-IRES-Cre; Ai148(TIT2L-GC6f-ICL-tTA2)	25	91	609	601
Vip;Ai148	Vip-IRES-Cre; Ai148(TIT2L-GC6f-ICL-tTA2)	14	59	505	497
Pvalb;Ai162	Pvalb-IRES-Cre; Ai162(TIT2L-GC6s-ICL-tTA2)	7	39	299	299

The number of mice, imaging sessions, neurons recorded, and neurons analyzed for each transgenic line in this study. The abbreviations for each line as used in the text and figures are also provided.

## Materials and Methods

### Dataset

We used the 2-photon calcium imaging recordings from the Allen Brain Observatory Visual Coding dataset (de Vries et al., 2020; Allen Brain Observatory – Visual Coding: Overview, 2018; Allen Brain Observatory – 2-Photon Visual Coding, 2016). This open dataset consists of neural activity recorded using 2-photon calcium imaging of transgenically expressed GCaMP6, surveying activity from neurons found in 6 cortical areas, 4 cortical layers, and 14 transgenic Cre lines. Awake, head-fixed mice were imaged while viewing a diverse set of visual stimuli including drifting gratings, static gratings, natural scenes, natural movies, locally sparse noise, and a mean luminance gray screen (“spontaneous activity”). These stimuli were divided across three ∼1 h imaging sessions, and each field of view was imaged across 3 d to record these distinct sessions. A set of three sessions from a given field of view is an “experiment container.” Cell matching was performed to match identified ROIs across the sessions within an experiment container. The mice were habituated to head fixation and to the visual stimuli used in the experiments, including one 60 min habituation session on the imaging rig itself, >2 weeks before data collection. Neural recordings were made from the left hemisphere while the mouse viewed a monitor to its right side. To track the eye movements of the mouse, the right side of the mouse was illuminated with an infrared LED and the right eye was imaged using an Allied Vision camera (30 frames/s; model Mako G-032B with GigE interface). All experimental procedures are detailed in the Allen Brain Observatory Visual Coding technical white paper (Allen Brain Observatory – Visual Coding: Overview, 2018). Table 1 lists the transgenic lines in the datasets and the number of experiment sessions analyzed in this study for each line.

### Stimulus abbreviations

LSN-4 and LSN-8 refer to locally sparse noise, 4° and 8°, respectively. SGs refers to static gratings and DGs refers to drifting gratings. NM-1, NM-2, and NM-3 refer to natural movies 1, 2, and 3, respectively, and NSs refers to natural scenes. The stimulus parameters are detailed in de Vries et al. (2020).

### Eye-tracking methods

The eye position was imaged on the eye that was viewing the visual stimulus. Details on the setup of the rig and the geometry of the eye-tracking apparatus are given in the Allen Brain Observatory Visual Coding technical white paper (Allen Brain Observatory – Visual Coding: Overview, 2018).

### Analysis

All analyses were performed using custom scripts written in Python 3.7, using NumPy (Harris et al., 2020), SciPy (Virtanen et al., 2020), pandas (McKinney, 2010), Matplotlib (Hunter, 2007), and the Allen SDK.

### Eye video trace extraction

We trained a single, universal eye-tracking model in DeepLabCut (Mathis et al., 2018), a ResNET-50-based network, to recognize up to 12 tracking points, each, around the perimeter of the eye, the pupil, and the corneal reflection, respectively. We used a published numerical routine to fit ellipses to each set of up to 12 tracking points (see Zenodo, bdhammel/least-squares-ellipse-fitting: v2.0.0: https://doi.org/10.5281/zenodo.3723294). For each ellipse, we reported the following parameters: *x*- and *y*-coordinates of the ellipse center, the width and height half-axis of the ellipse, and the rotation angle ϕ around the center. Fits were performed individually on each frame if there were at least six tracked points with a confidence of at least 0.8 as reported by the output of DeepLabCut. For frames where there were fewer than six tracked points, the ellipse parameters were set to not-a-number (NaN).

The training dataset contained two sources of hand-annotated data. We uniformly sampled 40 (of 1848) behavior movies at random and extracted three frames from each (for a total of 120 frames) via the *kmeans* option in DeepLabCut for annotation. On each frame, eight points each were annotated, approximately equidistantly around the eye and pupil, respectively. The center of the corneal reflection was annotated with a single point. These labeled frames comprised dataset 1. Next, we manually annotated 4150 frames with a rotated ellipse fitting the pupil and a point for the corneal reflection; the perimeter of the eye was not annotated. These labeled frames formed dataset 2. Then, to obtain a training dataset where 12 tracking points were annotated for each of the eye, the pupil, and the corneal reflection, respectively, we bootstrapped the above training data as follows. We trained a model on dataset 1 and used it to track the eye outlines on the data in dataset 2. This yielded 4150 frames fully annotated with ellipses (human annotation for pupil and corneal reflection, DeepLabCut annotation for the eye). Twelve points, in angular increments of 30° were computed along each fitted ellipse for use as training dataset 3. A new model was trained on this new dataset 3; 100 frames were extracted from each of the 40 videos used to create dataset 1, and the new model was used to fit 3 × 12 points to the 4000 frames obtained in this manner yielding dataset 4. The datasets 3 and 4 were combined, and the final, universal eye-tracking model was trained on the combined 8150 frames for 1,030,000 iterations (the DeepLabCut default maximum number of iterations).

### Angular eye position measurements

We first computed the corneal reflection position vector in the eye reference frame by assuming that the eye acts as a spherical mirror with radius 0.1682 cm and identifying the eye to infrared LED position vector. Next, for each video frame, we used the pupil and corneal reflection fit points predicted by DeepLabCut to compute the 2d position (in cm) in the monitor plane, achieved by projecting the pupil location fit points (translated into the eye reference frame by aligning the predicted corneal reflection to the aforementioned corneal reflection position vector) onto the monitor plane (also relative to the eye position frame) defined by the position of the monitor relative to the eye (15 cm; note that the monitor was not rotated relative to the eye, hence its normal vector pointed toward the center of the eye). We finally used this projection to compute the pupil position on the monitor in units of degrees, where the azimuth *a* was computed according to tan(a)=x/d and the elevation e was computed according to $\text{tan}\left(e\right)=\text{y}/\sqrt{{\text{x}}^{2}+{\text{d}}^{2}}$, where x and y are the projected eye position on the monitor plane and d is the distance from the eye to the monitor. These methods were performed in Python using the “eye_calibration” module of the Allen SDK, which has been previously published (de Vries et al., 2020).

### Automated detection of saccades

Using the extraction pipeline detailed above, we computed the absolute movement speed (in °/s) using the arc lengths between successive eye-tracking frames. Following a similar approach as in previous studies (Ramirez and Aksay, 2021), we defined the eye speed outlier threshold as μ + 3σ or 10°/s, whichever was higher (where statistics are computed in the entire trace during the experiment, ignoring NaN frames), and identified all of the frames during the experiment when the eye speed exceeded this threshold. Around each of these frames, we identified the frames where the speed exceeds μ + 1σ, resulting in a start and end frame for each saccade.

To remove saccades that were flagged incorrectly due to noisy position traces, eye bulging, or other animal jitter, we computed the mean and SD of the eye position trace in the 300 ms before saccade onset and 300 ms after saccade offset, for both azimuth and elevation. We ignored saccades that had any dropped data in these traces. We then computed two thresholds of $3\cdot \max\left({\text{σ}}_{\text{before}},\;{\text{σ}}_{\text{after}}\right)$ along these two dimensions to quantify the amount of noise in the trace; a larger quantity indicated a more substantial amount of noise in the trace. We computed the absolute difference in eye position during the flagged saccade, and the saccade was only considered valid if this difference exceeded the threshold in at least one dimension.

We computed the direction of each saccade by comparing the eye position at the saccade offset to the position at the onset. If the angle fell in the 90° sector between 45° and 315° (i.e., between −π/4 and π/4), the direction was set as temporal (right); if the angle fell in the 90° sector between 135° and 225° (i.e., between 3π/4 and 5π/4), the direction was set as nasal (left).

### Saccade analysis

From each experimental session, we identified which visual stimulus was present during each saccade. Since each individual mouse was imaged in one to three sessions, we aggregated the saccade frequency per stimulus statistic across these multiple sessions by dividing the total number of saccades made during a stimulus by the total duration the mouse was shown the stimulus.

### Data inclusion criteria

To analyze the saccade responses, we combined the experiment sessions from each experiment container to use all saccades for each neuron. Saccades that were not preceded by at least 2 s of steady gaze (i.e., no saccades) were excluded. Experiment containers with <15 nasal saccades or <15 temporal saccades were excluded. Of the dataset containing 43,316 neurons, we analyzed data from 32,442 neurons across 205 mice and 818 sessions (Table 1).

### Saccade visual activity modulation

To compare our results with recent work done by Miura and Scanziani (2022), we performed two analogous tests to identify neurons with significantly modulated responses. This analysis was done using the dF/F computed from the raw calcium fluorescence traces, as described in de Vries et al. (2020). The first test was a Wilcoxon signed-rank test comparing the mean dF/F after the saccade onset (frames 0–10 relative to the saccade onset; i.e., 0–333 ms after saccade start) to the mean dF/F before the saccade (frames −20 to −10 relative to the saccade onset; 667–333 ms before saccade start). The second test was a Wilcoxon rank-sum test comparing the mean dF/F after the saccade onset (same range) for saccades in nasal versus temporal directions. The significance threshold on both tests was set as p = 0.05, after adjusting for multiple comparisons by setting the false discovery rate to 10% using the Benjamini–Hochberg procedure. A neuron was considered to have significantly modulated visual activity if there was significance in at least one of the two tests. Note that this is different from our saccade responsive definition, which is described below.

### Neural activity bootstrapping

We used bootstrapping methods to compute percentiles and *p*-values to quantify the responsiveness of neurons to saccades. We used dF/F traces provided by the Allen Brain Observatory (de Vries et al., 2020). For every neuron, we computed the average dF/F in frames 0–10 (0–333 ms) relative to the saccade onset for each saccade, along with the average dF/F in frames −45 to −15 (−1.5 to −0.5 s) relative to the saccade onset. We then subtracted the latter from the former, yielding the change in dF/F value for each neuron and saccade. We computed the mean change in dF/F for each neuron, referred to as its mean saccade response, by averaging the values across all saccades.

We used this same response computation to build a bootstrapped baseline distribution of containing *N* = 40,000 samples for each neuron, where each sample was computed using the same procedure above, except the onset times were determined using the following procedure: (1) randomly choose an experiment session among the set of sessions in which a neuron was present, where the sessions are weighted by their total number of saccades; and (2) select a frame uniformly at random across the entire chosen session. We then computed the quantile q (expressed as a value between 0 and 1) of the mean saccade response of a neuron within the bootstrap distribution. Specifically, if q was within a *p*-value of 1, we flagged the neuron as having an enhanced response to saccades; if q was within a *p*-value of 0, we flagged the neuron as having a suppressed response to saccades; and otherwise we flagged the neuron as having no response to saccades. SR neurons were those that had either an enhanced or suppressed response to saccades. We used $p\text{ = 5 }\times 10^{-4}$, which from our sample of ∼32,000 neurons would give ∼16 false positives.

### Direction-selective inclusion criteria

Using a procedure analogous to that used in the study by Miura and Scanziani (2022), we compared the average dF/F in frames 0–10 relative to the saccade onset in nasal and temporal directions using a Wilcoxon rank-sum test, and flagged an SR neuron as DS if the result of this test was significant (*p* < 0.05). For DS neurons, we defined the preferred direction as the direction (nasal or temporal) that maximized the mean saccade response of that neuron.

### Spontaneous saccade inclusion and bootstrapping

To compare our results to an alternate SR neuron definition that only considers saccades made during the spontaneous stimulus (mean luminance gray screen), we applied an analogous procedure as in the subsection Data inclusion criteria, the only differences being that we included only saccades that occurred during the spontaneous activity and used a threshold of at least three nasal and at least three temporal saccades for each neuron. Using different thresholds did not impact the results, and this threshold was chosen to consider a comparable number of neurons as shown previously (*n* = 30,764 of 43,316 under this criterion). We then applied procedures analogous to those in the subsections Neural activity bootstrapping and Direction-selective inclusion criteria, with the only differences being that our null distribution was composed of only neural responses during the spontaneous activity epoch.

### Saccade neural responses to different visual stimuli

To see how the response of a saccade-responsive neuron varied depending on different stimuli, we first considered the saccade response classification of a neuron: if the neuron was responsive to saccades in a direction-selective manner, then we considered only saccades in the preferred direction; otherwise, we considered saccades in all directions. We excluded experimental sessions containing fewer than eight saccades during the spontaneous stimulus. For each neuron, we computed the response to each saccade using the same procedure as in Materials and Methods, in the subsection Neural activity bootstrapping. We used a Wilcoxon rank-sum test on related paired samples (namely, the mean responses to saccades made during spontaneous stimulus and the mean responses to saccades made during the other compared visual stimulus). We computed normalized responses for each session by dividing the mean response of a neuron for saccades made during each stimulus by its mean response to saccades made during the spontaneous stimulus.

### Visual response metrics

To assess the visual responses of SR neurons, we used response metrics previously computed for the dataset and described in de Vries et al. (2020). For the response of each neuron to drifting gratings, the preferred condition was identified as the grating direction and temporal frequency at which the neuron had its largest mean response. This preferred condition determined the preferred direction of a neuron.

Direction-selectivity index (DSI) for each neuron was computed as follows using its mean response to the drifting gratings stimulus at its preferred temporal frequency:

$$\text{DSI}=\frac{R_{\text{pref}}-R_{\text{null}}}{R_{\text{pref}}+R_{\text{null}}},$$

where $R_{\text{pref}}$ is the mean response of a neuron to its preferred direction, and $R_{\text{null}}$ is its mean response to the opposite direction at the same temporal frequency.

Orientation-selectivity index (OSI) was computed from the mean response to the drifting gratings stimulus at the preferred temporal frequency of each neuron as follows:

$$\text{OSI}=\frac{\sum^{}R_{\theta }e^{2i\theta }}{\sum^{}R_{\theta }},$$

where $R_{\theta }$ is the neuron’s mean response to direction θ (Ringach et al., 1997).

The lifetime sparseness (*S_L_*) was computed using the definition in Vinje and Gallant (2000), as follows:

$$S_{L}=\frac{1-\frac{1}{N}\frac{{\left(\sum_{i}r_{i}\right)}^{2}}{\sum_{i}r_{i}^{2}}}{1-\frac{1}{N}},$$

where N is the number of stimulus conditions and $r_{i}$ is the response of the neuron to stimulus condition i averaged across trials.

### Data availability

The code/software described in the article is freely available online at https://github.com/AllenInstitute/visual-coding-saccades.

## Results

### Characteristics of saccades in head-fixed mice during diverse visual stimuli

**Figure 1.**
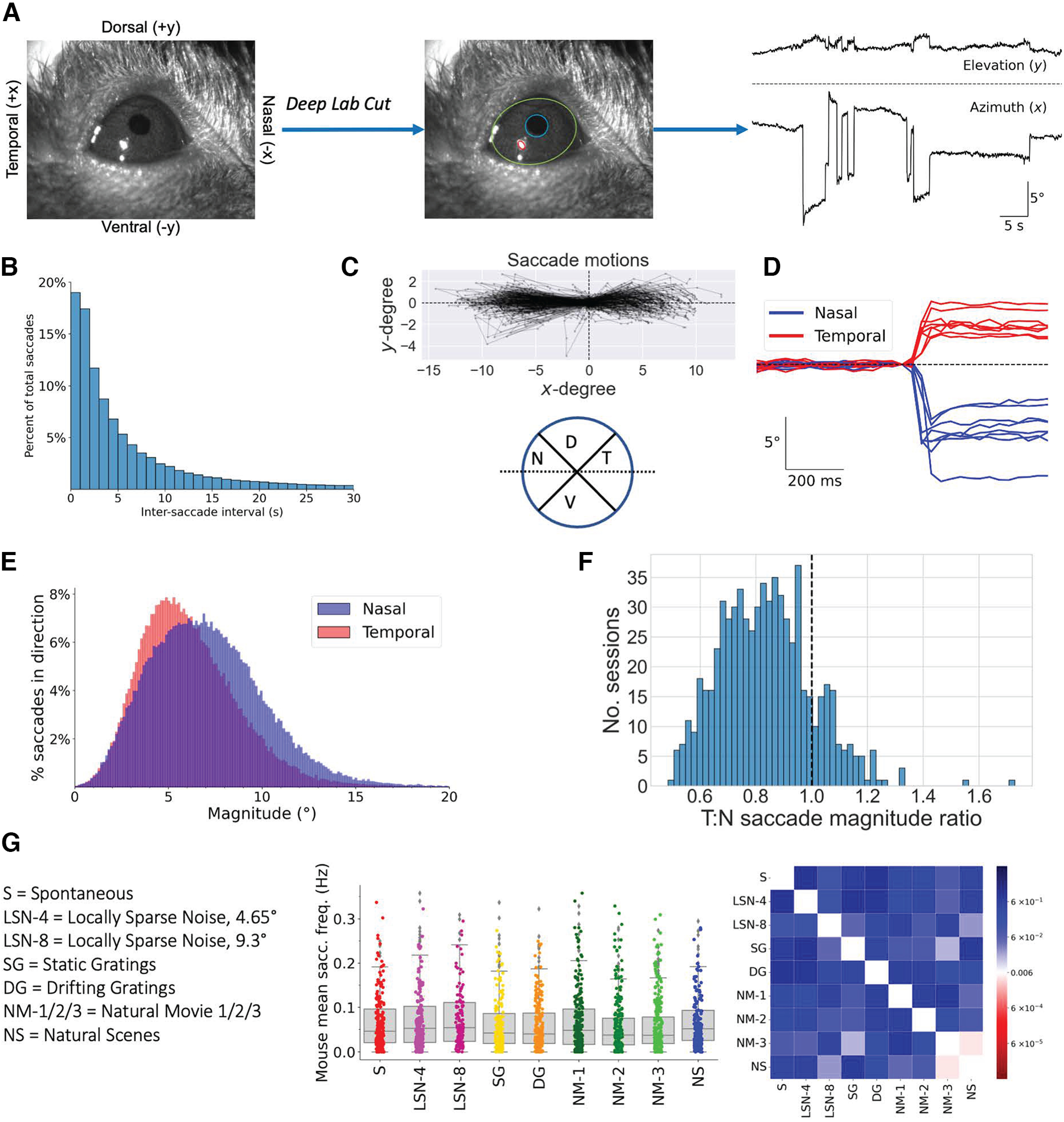
Saccade behavior is not influenced by visual stimulus. ***A***, Left, Example frame from eye video annotated with axes of eye movement (*x*-axis, nasal–temporal; *y*-axis, dorsal–ventral). Middle, Example ellipse fits on eyeball (green), pupil (blue), and glare spot (red) from DeepLabCut model (Materials and Methods). Right, Example azimuth (horizontal) and elevation (vertical) traces from an experiment session after pupil extraction (Materials and Methods). Top trace is the elevation (*y*-degree, vertical), bottom trace is the azimuth (*x*-degree, horizontal), and dashed line indicates 0°. Upward is positive, and downward is negative using the same signs as in the left subplot. Times of abrupt eye position change correspond to saccades. ***B***, Intersaccade intervals (s), defined as the time elapsed before the preceding saccade, for all saccades. Of *n* = 202,156 total saccades, 33.5% saccades occurred at most 2 s after a previous saccade. ***C***, Top, Example saccade motions from one experiment session, where each saccade has been aligned to start at the origin. Each saccade is a separate line, and points on the line indicate eye position at frames during the saccade. Note that saccades are made preferentially in the horizontal direction. Bottom, Direction classification of saccades. Each sector of the circle is 90°; dashed black line indicates the horizontal axis. Middle intersection point denotes eye position alignment of the start of saccade. T, Temporal; D, dorsal; N, nasal; V, ventral. ***D***, Example nasal and temporal saccades from one experiment session. Dashed line represents 0°, and traces are centered by subtracting the position at the time of saccade onset. Only azimuthal (horizontal) traces are shown. ***E***, Distributions of saccade magnitude for nasal and temporal saccades. Nasal saccades (*n* = 104,121), 7.10 ± 3.19° (mean ± SD); temporal saccades (*n* = 91,037), 6.14 ± 2.78°. Grouped together, all horizontal saccades (*n* = 195,158) had magnitude 6.65 ± 3.05°. Density normalization accounts only for saccades in the given direction (i.e., *y*-axis is normalized so both nasal and temporal area curves have unit area). ***F***, Ratio of mean temporal saccade magnitude to mean nasal saccade magnitude in each individual session. Dashed vertical line at 1, indicating an equal magnitude of nasal and temporal saccades. ***G***, Left, Visual stimuli abbreviations used in this figure and throughout the article. Middle, Saccade frequency for different visual stimuli. Each data point is the average saccade frequency of a mouse for a particular visual stimulus (Materials and Methods). Box line corresponds to the 25th to 75th percentile, line corresponds to median (50%), and error bars at 10% and 90%. Right, Probability heatmap matrix comparing mouse mean saccade frequencies across different stimulus types using a two-sample KS test. Heatmap is centered at Bonferroni-corrected significance value (*p* = 0.05/8 ≅ 6e-3).

To better understand how mice in head-fixed contexts make saccadic eye movements, we extracted the eye position from eye-tracking videos recorded during simultaneous stimulus presentation on a monitor placed contralateral to the recorded hemisphere of the mouse (de Vries et al., 2020). Specifically, we used DeepLabCut (Mathis et al., 2018) to fit ellipses to the eye and pupil in each frame of the videos, from which we calculated the pupil position in degrees (Fig. 1*A*; Materials and Methods). From this, we identified the occurrence of saccades by finding consecutive frames where the eye speed exceeded a threshold (Materials and Methods).

Across 818 1 h experimental sessions, we identified a total of 202,346 saccades. The distribution of time elapsed between successive saccades had a long right tail, consistent with previous results that suggest saccades often occur in bursts separated by longer periods of fixation (Fig. 1*B*; Samonds et al., 2018). Specifically, approximately one-third (33.5%, *n* = 67 506) of all saccades were preceded by another saccade within 2 s.

We classified the direction of a saccade based on the direction of eye movement (Fig. 1*C*,*D*). Consistent with other studies of head-fixed mice (Samonds et al., 2018; Meyer et al., 2020; Miura and Scanziani, 2022), we found that saccades were made almost exclusively along the horizontal axis. Specifically, 96.6% (*n* = 195,477) of all saccades ended within 45° from horizontal, with slightly more made in the nasal direction (nasal: 51.5%; *n* = 104,202; temporal: 45.0%; *n* = 91,245). Also consistent with previous studies in both freely moving (Meyer et al., 2020) and head-fixed (Sakatani and Isa, 2007; Itokazu et al., 2018; Miura and Scanziani, 2022) mice, we observed a systematic asymmetry in the magnitudes of nasal (mean ± SD; 7.10 ± 3.19°) and temporal (6.13 ± 2.79°) saccades [Fig. 1*E*; mean ± SD; *p* = 0.0, two-sample Kolmogorov–Smirnov (KS) test]. This discrepancy in magnitude was also found within individual sessions: the ratio of mean temporal saccade magnitude to mean nasal saccade magnitude within individual sessions was 0.84 ± 0.17, suggesting further that nasal saccades are slightly larger in magnitude than temporal saccades (Fig. 1*F*).

Given that the visual coding dataset records neural responses to a variety of visual stimuli—including DGs; SGs; LSN; NSs; NM-1, NM-2, and NM-3; and spontaneous activity (S)—we considered whether mice might make more saccades during certain visual stimuli by comparing the saccade frequencies during each of the visual stimuli (Fig. 1*G*). A recent study found that a using a single LED light as a visual stimulus did not change the probability of saccade generation (Zahler et al., 2021), and the more complex stimuli that we investigated here yielded heavily overlapping distributions of saccade frequency, indicating that visual stimuli do not heavily influence the saccade frequency. The only pairing of statistically significant distributions was between NSs and NM-3, suggesting that mice may make slightly more eye movements during NS compared with NM-3. Given that there are no significant differences between in saccade distributions during NS and NM-1 or NM-2, however, it seems unlikely that this a meaningfully robust result.

### Neurons in visual cortex respond to saccades

To identify saccade-responsive neurons, we examined the dF/F traces of neurons surrounding each saccade. Our analysis included 32,442 neurons imaged in one or more imaging sessions (Materials and Methods). To compare our data with previous studies (Itokazu et al., 2018; Miura and Scanziani, 2022), we performed analogous tests to identify neurons with significantly modulated activity following saccades, directly comparing the dF/F activity following each saccade with activity preceding each saccade (Materials and Methods). Using these tests, we found that 17,109 of 32,442 neurons (52.7%) had significantly modulated activity following saccades, an estimate that is consistent with this previous work.

**Figure 2.**
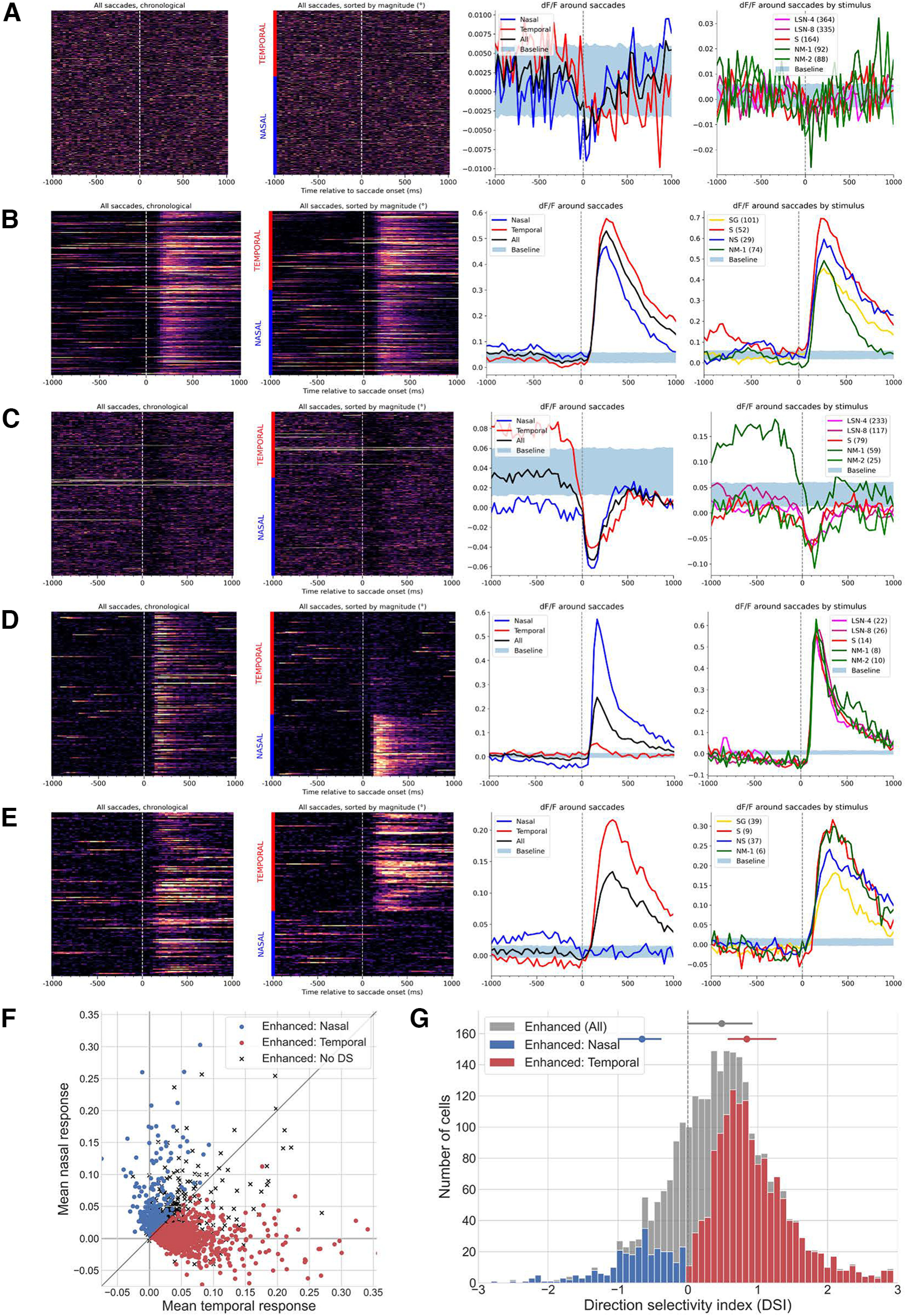
Diverse responses of neurons in visual cortex to saccades. ***A***, Example neuron with a significant activity modulation (Materials and Methods; *p* = 0.0024, Wilcoxon signed-rank test). Neuron 669928467 (Tlx3;Ai148, VISl, L5, session 658854486). Left, Each row is a dF/F trace centered around a saccade, where saccades are ordered in chronological order (top corresponds to the start of the experiment) and time 0 corresponds to the start of each saccade. Second from left, All temporal and nasal saccades (indicated by red and blue bars), where each group is sorted by magnitude (largest magnitude on top). Third from left, Mean dF/F of neuron around saccades. Nasal (blue) and temporal (red) traces are mean dF/F around nasal and temporal saccades, respectively; all (black) corresponds to dF/F average around all saccades. Baseline is a *N* = 1000 bootstrapped distribution where each trace is the mean of random time points within the experiment session (the number of random samples equals the number of saccades). Right, dF/F average around preferred saccades by stimulus type. Baseline is the distribution from the previous subplot; the number in the legend is the number of preferred saccades during each stimulus. ***B***, Example SR neuron with an enhanced response to saccades, regardless of direction. Neuron 517476630 (Cux2;Ai93, VISal L2/3, session 506156402). ***C***, Example SR neuron with a suppressed response to saccades, regardless of direction. Neuron 670074250 (Cux2;Ai93 VISrl L2/3, session 662960692). ***D***, Example SR, DS neuron preferring nasal saccades. Neuron 662076627 (Ntsr1;Ai148, VISp L6, session 606227591). ***E***, Example SR, DS neuron preferring temporal saccades. Neuron 662209107 (Ntsr1;Ai148, VISp L6, session 636889229). ***F***, Scatter plot of saccade-responsive enhanced neurons showing their mean temporal saccade response (*x*-axis) and mean nasal saccade response (*y*-axis). Blue dot indicates that a neuron prefers nasal saccades; red dot indicates that neuron prefers temporal saccades; black “x” indicates the neuron is not direction selective. Mean nasal/temporal response for nasal neurons, 0.0528/0.0074; temporal neurons, 0.0015/0.0490; non-direction-selective neurons, 0.0249/0.0302. See Extended Data Figure 5-1*A* for distribution where SR neurons are detected using saccades during spontaneous visual stimulus. ***G***, Direction selectivity index histogram for saccade-responsive enhanced neurons. Gray, All saccade-responsive enhanced neurons (*n* = 2696); blue, neurons that prefer nasal saccades (*n* = 318); red, neurons that prefer temporal saccades (*n* = 1467). Bar is the middle 50% (all: −0.01, 0.93; nasal: −1.02, −0.39; temporal: 0.58, 1.27); dot is the median (all, 0.49; nasal, −0.68; temporal, 0.84). See Extended Data Figure 5-1*A* for distributions where SR neurons are detected using saccades during spontaneous visual stimulus.

However, because of the high variability of neural activity over time, it is possible for activity to have statistically significant differences in presaccade versus postsaccade time intervals, but to fail to achieve significance when compared with randomly located moments in time. Indeed, from visual inspection it was clear that many of these modulated neurons did not exhibit clear and robust enhanced (or suppressed) responses to saccades (Fig. 2*A*). Thus, to identify neurons with robust saccade responses, we used a stricter bootstrapping procedure to estimate the null distribution of dF/F changes at randomly sampled (with replacement) time points throughout the entire experiment (Materials and Methods). This procedure identified SR neurons across all cortical areas and layers that exhibited robust responses to eye movements in a variety of manners. Overall, 9.4% (*n* = 3068) of analyzed neurons were SR. The saccade responses of SR neurons fell primarily into four distinct categories. First, 33.9% (*n* = 916) of SR neurons exhibited a strong enhanced (positive) response to all saccades, regardless of their direction (Fig. 2*B*). Second, 12.0% (*n* = 368) of SR neurons exhibited a strong suppressed (negative) response to all saccades (Fig. 2*C*). Finally, other SR neurons exhibited an enhanced response to saccades made along either the nasal or temporal direction, but no (or a slightly suppressed) response to saccades in the opposite direction (Fig. 2*D*,*E*; nasal: 10.1%; *n* = 310; temporal: 48.0%; *n* = 1474). We refer to such neurons as DS and define the direction—either nasal or temporal—that maximizes their response to be their preferred direction.

In our analysis, SR neurons were classified as DS if they met the following two criteria: (1) they have an enhanced response to saccades; and (2) they have a statistically significant difference in response to nasal and temporal saccades (Materials and Methods; *p* < 0.05, Wilcoxon rank-sum test). A DS neuron was further classified as preferring temporal saccades if T > N, where T was its mean response to temporal saccades and N was the mean response to nasal saccades, or as preferring nasal saccades if N > T (Fig. 2*F*). To quantify direction preference, we calculated a DSI metric for DS neurons, defined as (T−N)/(T+N) (Fig. 2*G*). Note that, given that T and N can take on negative values, our DSI metric is not necessarily constrained between −1 and +1; furthermore, a positive DSI indicates a temporal saccade preference, and a negative DSI indicates a nasal saccade preference. The mean DSI of temporal saccade-preferring neurons was 1.08 ± 2.37, and that of nasal saccade-preferring neurons was –0.86 ± 0.99 (mean ± SD). Surprisingly, we observed a strong bias for DS neurons to prefer temporal saccades over nasal saccades (temporal, *n* = 1474 neurons; nasal, *n* = 310 neurons), a result that has not been discussed in prior saccade literature. Applying the DSI computation to all enhanced SR neurons, we found the mean to be 0.53 ± 1.98, suggesting that even non-DS SR-enhanced neurons responded more strongly on average to temporal saccades than nasal saccades. This bias was not because of a discrepancy in the quantity of nasal and temporal eye movements within sessions, as 47.6 ± 12.9% (mean ± SD) saccades in each experiment were nasal, and 47.2 ± 11.5% were temporal. This even split between nasal and temporal saccades suggests that the bias toward neurons preferring temporal saccades is a result of neural dynamics rather than behavioral tendencies.

**Figure 3.**
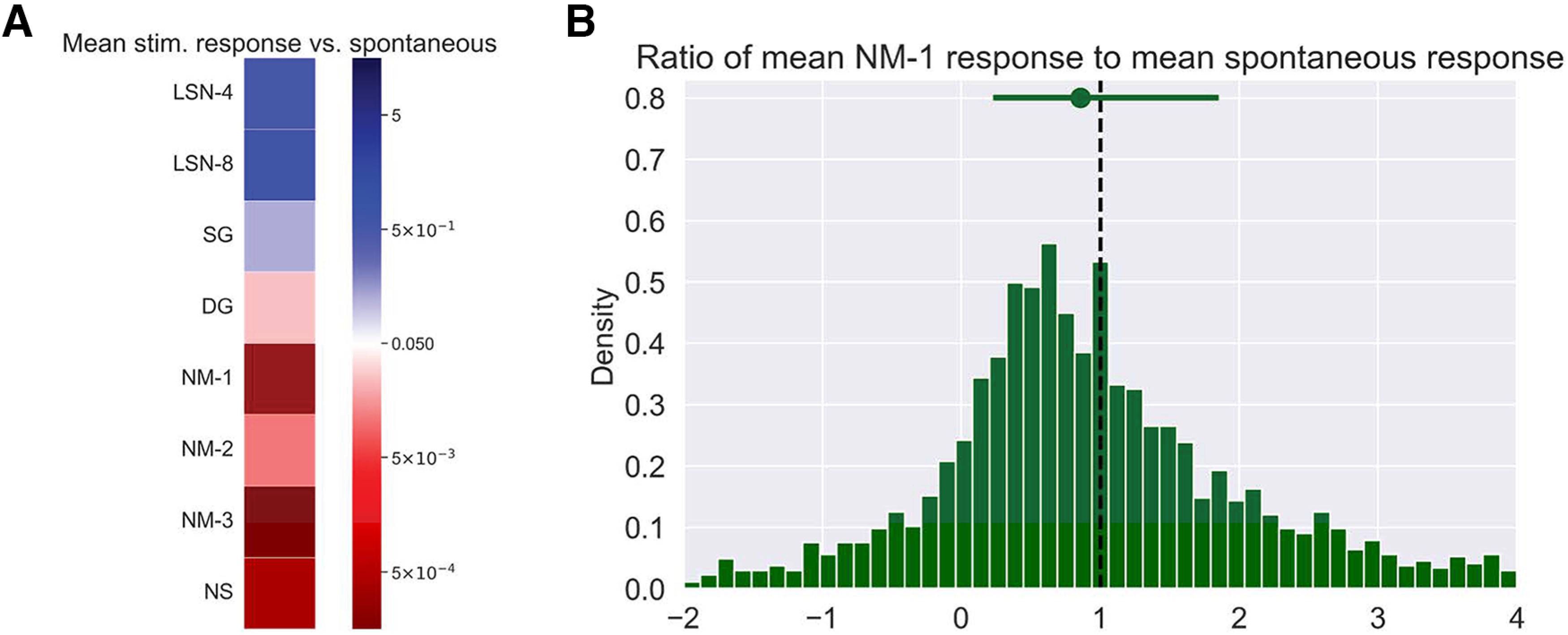
Saccade responses are more strongly suppressed by naturalistic stimuli. ***A***, Comparison of mean response to saccades made during spontaneous visual stimulus and other visual stimuli (Materials and Methods). Significance value is *p* = 0.05 using Wilcoxon rank-sum test. ***B***, Ratio of the mean response to preferred saccades made during NM-1 to the mean response of preferred saccades made during spontaneous for each saccade-responsive neuron. Vertical black dashed line at 1 indicates equal response. Green dot is the median ratio, bars extend to 25% and 75%. See Extended Data Figure 3-1 for an analogous plot for each visual stimulus.

How individual SR neurons vary their responses during visual stimulation might inform alternative models for how cortex integrates saccades in the presence of visual stimuli. Generally, SR neurons exhibited strong responses to saccades independent of visual stimulus, but the degree of that response for particular neurons was sometimes enhanced or suppressed by different visual stimuli. To quantify this variability, we measured the saccadic response during particular visual stimuli relative to the size of the saccadic response during a mean luminance gray screen (the “spontaneous” stimulus). We only considered experimental sessions where at least eight saccades were made during the spontaneous stimulus (Materials and Methods). Furthermore, for DS neurons, we investigated only those saccades made in the preferred direction of a neuron. We first compared the mean saccade response to saccades made during the spontaneous stimulus to the mean saccade response during the other visual stimuli (Fig. 3*A*; Wilcoxon signed-rank test). We found no significant difference in saccade responses during static artificial stimuli (LSN-4, LSN-8, SG) compared with responses to saccades made during the spontaneous stimulus. However, we observed significant differences in the saccade responses between spontaneous and moving or naturalistic stimuli (DG, NM-1, NM-2, NM-3, and NS), suggesting more substantial heterogeneity in saccade responses during these visual stimuli. To better understand how these responses varied by stimulus, we compared the ratio of the mean response to saccades made during NM-1 to the mean response to saccades made during spontaneous stimulus (Fig. 3*B*, Extended Data Fig. 3-1). While there was considerable variation in these ratios, the median ratio for every stimulus was <1 (median values: LSN-4, 0.79; LSN-8, 0.79; SG, 0.76; DG, 0.81; NM-1, 0.86; NM-2, 0.78; NM-3, 0.81; NS, 0.76), suggestive of a diminished response to saccades made during these dynamic or naturalistic visual stimuli when compared with the response to saccades made during a gray screen. This indicates that while there is great heterogeneity in saccade responses of SR neurons, these responses are marginally suppressed during naturalistic—but not artificial—stimuli. Recalling how we define a saccadic response—the difference in neural activity around the saccade—the observed diminished saccade response during natural stimuli may in part be caused by elevated cortical activity and increased responsiveness of neurons during natural versus artificial stimuli (de Vries et al., 2020).

10.1523/ENEURO.0051-23.2023.f3-1 Figure 3-1 Ratio of the mean saccade response during each stimulus to the mean response during gray screen. Dashed vertical line at *x* = 1 indicates an equal mean response. Top bar and dot represent middle 50% and median, respectively. Download Figure 3-1, TIF file.

### Differences in saccade response across transgenic lines and cortical areas

**Figure 4.**
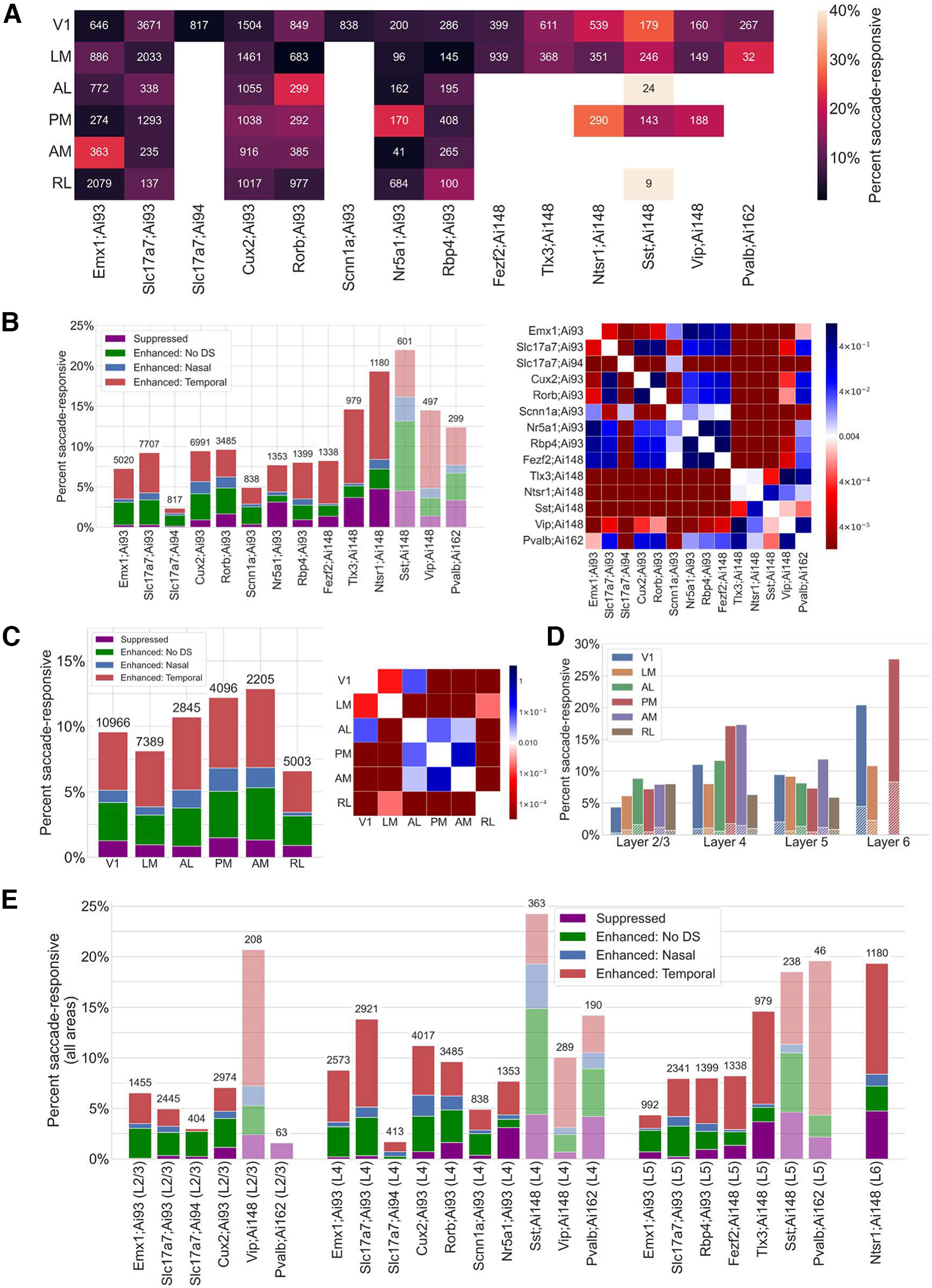
Differences in saccade responses across transgenic lines and visual areas. ***A***, Heatmap of the percentage of SR neurons by transgenic line and visual area, relative to the total number of imaged neurons, which is given above each bar. ***B***, Left, Percentage of SR neurons by transgenic line, broken down by type of response. “Enhanced: No DS” means that the neuron has an enhanced response to saccades but does not prefer any one direction (Materials and Methods). Number above each bar is the total number of imaged neurons of the corresponding line. Low-opacity bars indicate inhibitory neurons. Right, χ^2^ test across pairings of transgenic lines (using a 2 × 2 contingency matrix containing number of SR and non-SR neurons for each line). ***C***, Left, Percentage of SR neurons by visual area. Number above each bar is the total number of imaged neurons in the corresponding area. Right, Heatmap; same as in ***B***. ***D***, Percentage of SR neurons by cortical layer and visual area. Hatched marks indicate neurons with a suppressed saccade response. ***E***, Percentage of SR neurons by transgenic line and cortical layer, across all visual areas. Low-opacity bars indicate inhibitory neurons, and the numbers above bars indicate the total number of neurons imaged. See Extended Data Figure 4-1 for an analogous plot for neurons only in V1.

The scale of the Allen Brain Observatory dataset enables us to compare saccade responses across neurons in different visual areas, transgenic lines, and cortical layers (Table 1). Saccade-responsive neurons were found across all visual areas, transgenically defined cell types, and cortical layers— though not at the same frequency across these dimensions (Fig. 4*A*). Sst;Ai148 neurons exhibited the highest overall SR frequency, at 22.0% (132 of 601), whereas Slc17a7;Ai94 neurons exhibited the lowest overall SR frequency, at 2.3% (19 of 817). We observed higher proportions of both SR and SR-suppressed neurons in TIGRE 2.0 (Ai148 or Ai162; Daigle et al., 2018) reporters than the TIGRE 1.0 (Ai93 or Ai94; Madisen et al., 2015) reporters (Fig. 4*B*). As there is no Cre diver that was used with both TIGRE 1.0 and TIGRE 2.0 constructs, however, we are unable to determine whether this difference is because TIGRE 2.0 was used specifically for inhibitory neurons and excitatory neurons in deep layers, or whether this is the result of an off-target effect of the overexpression of either tTA or GCaMP using the TIGRE 2.0 reporter. Additionally, DS SR neurons exhibited a noticeably higher preference for temporal saccades over nasal saccades, and this observation was consistent across all lines.

10.1523/ENEURO.0051-23.2023.f4-1 Figure 4-1 Percentage of saccade-responsive neurons in V1. Percentage of SR neurons by transgenic line and cortical layer, across only V1. Low-opacity bars indicate inhibitory neurons, and the numbers above bars indicate the total number of neurons imaged. See Extended Data Figure 5-1 for an analogous figure where SR neurons are detected using saccades during the spontaneous visual stimulus (Materials and Methods). Download Figure 4-1, TIF file.

We also observed high percentages of saccade-responsive neurons in anterolateral (AL), anteromedial (AM), and posteromedial (PM) areas (Fig. 4*C*), and these rates were consistent across these three areas and statistically different from other areas (χ^2^ test, *p* < 0.01). This higher rate of SR neurons in these dorsal areas is consistent with dorsal areas representing motion information or egocentric reference frames (Andermann et al., 2011; Marshel et al., 2011; Wang et al., 2012). Rostrolateral (RL) neurons exhibited the lowest percentage of SR neurons, at 6.7% (336 of 5003). However, this discrepancy with the other dorsal areas above (which exhibited greater proportions of saccade responsive neurons) could reflect the challenges of accurate retinotopic targeting of RL neurons (de Vries et al., 2020; Siegle et al., 2021).

Next, we investigated the distribution of SR neurons by layer. Previous work has shown that saccade-induced neural responses increase in excitability and discriminability as a function of cortical depth (Miura and Scanziani, 2022). We similarly find variations in the percentage of SR neurons by layer, with high percentages for neurons in layer 6 (Fig. 4*D*). In this deep layer, Ntsr1;Ai148 neurons imaged in V1, LM, and PM exhibited the highest overall frequencies of saccade responsivity. Interestingly, a higher proportion of SR layer 6 neurons exhibited a suppressed response relative to more superficial layers. We also found a high frequency of SR neurons in layer 4 PM and AM neurons. In aggregate, only a small fraction of superficial layer 2/3 neurons was SR, and this was consistent across all visual areas.

Finally, as some transgenic lines were imaged in different layers, we examined this layer-dependent breakdown across different transgenic lines (Fig. 4*E*). Broadly, excitatory neurons in deeper layers (e.g., Tlx3;Ai148 in layer 5 and Ntsr1;Ai148 in layer 6) tended to be more saccade responsive, but inhibitory neurons did not exhibit this same bias toward deeper layers. Specifically, across all visual areas, 20.7% (43 of 208) of Vip;Ai148 inhibitory layer 2/3 neurons were SR, 25.6% (93 of 363) of Sst;Ai148 inhibitory layer 4 neurons were SR, and 21.7% (10 of 46) of Pvalb;Ai162 inhibitory layer 5 neurons were SR—these were the highest rates across any population in all layers. In addition to these inhibitory populations showing higher proportions of suppressed saccade responses, they also tended to prefer specific layers, with Vip;Ai148 preferring layers 2/3, Sst;Ai148 preferring layer 4, and Pvalb;Ai162 preferring layer 5. This layer preference was also prevalent in some excitatory populations, with the most notable difference occurring in layer 5. In this layer, 14.2% (139 of 979) of Tlx3;Ai148 neurons were SR, while only 8.1% (108 of 1338) of Fezf2;Ai148 were SR. Tlx3;Ai148 neurons are corticocortical projecting neurons (Gerfen et al., 2013), while Fezf2;Ai148 neurons are corticofugal (Guo et al., 2013), suggesting that the saccadic activity is differentially represented in these distinct pathways. These cell type dependencies were also consistent when examining primary visual cortex specifically (Extended Data Fig. 4-1), with a minor difference being that V1 layer 2/3 excitatory cells were slightly less likely to be saccade responsive when compared with rates across all visual areas.

### Saccade-responsive neurons have similar visual responses as non-saccade-responsive neurons

Given that visual cortex receives both bottom-up visual input from the eyes and top-down feedback from other areas, a key question about saccade responses is whether they are driven by the change in visual features because of the eye movement, motor signals, or a combination of both. To consider this, we compared the visual response properties of SR neurons with those of non-SR neurons. If saccade responses are primarily visually driven, we would expect SR neurons to exhibit visual tuning properties that are distinct from those of non-SR neurons.

**Figure 5.**
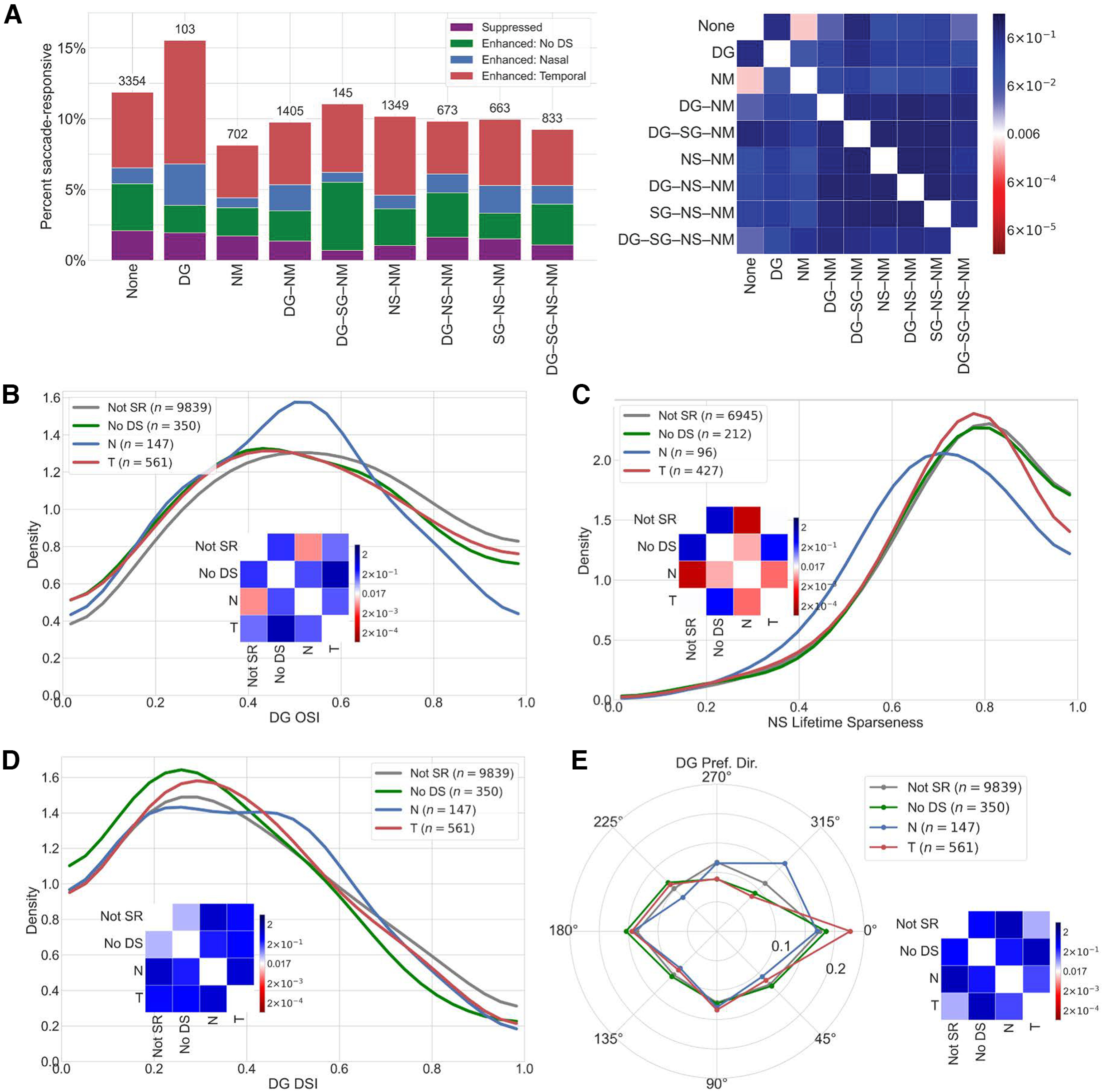
Saccade-responsive neurons have similar visual responses to non-saccade-responsive neurons. ***A***, Left, Percentage of SR neurons by visual response class, indicating which set of visual stimuli elicit a response. Number above each bar is the total number of imaged neurons within the given cluster. Right, χ^2^ test across pairings of transgenic lines (contingency matrix containing number of SR and non-SR neurons for each cluster; *p* = 0.05, Bonferroni corrected for multiple comparisons). ***B***, Orientation selectivity index for neurons that respond to drifting gratings. Curve is smoothed using a Gaussian kernel. Inset, Heatmap shows a KS test between different distributions (*p* = 0.05, Bonferroni corrected for multiple comparisons). ***C***, Analogous plot to ***B***, showing lifetime sparseness for neurons responsive to natural scenes. ***D***, Analogous plot to ***B***, showing a direction selectivity index plot for neurons responsive to drifting gratings. ***E***, Distribution of preferred direction for neurons responsive to drifting gratings. See Extended Figure 5-1 for an analogous figure where SR neurons are detected using saccades during the spontaneous visual stimulus (Methods).

Previous work had used a cluster analysis of the response reliabilities of neurons to four of the stimuli (DG, SG, NS, and NM - combining together NM-1, NM-2, and NM-3 responses) to identify functional response classes with this dataset (de Vries et al., 2020). This analysis categorized neurons based on the stimuli to which they responded reliably. Broadly, neurons fall into 9 of the possible 16 combinations of stimuli, including a “None” class in which neurons do not reliably respond to any of these visual stimuli. We examined how SR neurons were distributed across these functional classes and found that each of the nine classes was composed of ∼10% SR neurons (Fig. 5*A*). This observation that SR neurons are not biased toward a single functional response class suggests that SR neurons do not exhibit a particular visual response feature that is driven by saccades. We used a χ^2^ test with a 2 × 2 contingency table containing the number of SR and non-SR neurons in each cluster to compare these rates across classes, finding no significant differences (*p* >0.05, Bonferroni correction), except in one case between None and NM classes, suggesting that neurons responsive to no visual stimuli may be slightly more likely to respond to saccades than neurons responsive to only natural movies.

10.1523/ENEURO.0051-23.2023.f5-1 Figure 5-1 Spontaneous saccade-responsive neurons have similar visual responses to non-saccade-responsive neurons. Subplots A–E are analogous to those in Figure 5*A–E*, except that SR neurons in this figure are detected using saccades during spontaneous stimulus (Materials and Methods). ***A***, Left, Percentage of SR neurons by visual response class, indicating which set of visual stimuli elicit a response. The number above each bar is the total number of imaged neurons within the given cluster. Right, χ^2^ test across pairings of transgenic lines (contingency matrix containing the number of SR and non-SR neurons for each cluster; *p* = 0.05, Bonferroni corrected for multiple comparisons). ***B***, Orientation selectivity index for neurons that respond to drifting gratings. Curve is smoothed using a Gaussian kernel. Inset: Heatmap shows KS test between different distributions (*p* = 0.05, Bonferroni corrected for multiple comparisons). ***C***, Analogous plot to ***B***, showing lifetime sparseness for neurons responsive to natural scenes. ***D***, Analogous plot to ***B***, showing a direction-selectivity index plot for neurons responsive to drifting gratings. ***E***, Distribution of preferred direction for neurons responsive to drifting gratings. ***F***, Compare with Figure 2*F*. Scatter plot of saccade-responsive enhanced neurons showing their mean temporal saccade response (*x*-axis) and mean nasal saccade response (*y*-axis). Blue dot indicates that the neuron prefers nasal saccades, red dot indicates that neuron prefers temporal saccades, and black “x” indicates that the neuron is not direction selective. ***G***, Compare with Figure 2*G*. Direction selectivity index histogram for saccade-responsive enhanced neurons. Gray, All saccade-responsive enhanced neurons (*n* = 1,026); blue, neurons that prefer nasal saccades (*n* = 81); red, neurons that prefer temporal saccades (*n* = 342). The bar is the middle 50% (all: –0.11, 0.78; nasal, –1.08, -0.57; temporal, 0.66, 1.23); dot is the median (all, 0.34; nasal, –0.84; temporal, 0.89). Download Figure 5-1, TIF file.

Given that we observe no clear preference for functional visual response class among SR neurons, we sought to identify any differences in broader tuning metrics for SR neurons to different visual stimuli. While it would be interesting to compare the size and structure of the spatial receptive fields mapped with locally sparse noise, there unfortunately were not enough SR neurons with mapped receptive fields to draw meaningful conclusions [*n* = 342 of 1043 SR neurons, across all SR subtypes, with at least one subfield (on or off) in V1, and 380 of 2028 in higher visual areas]. For the cells that did have mapped receptive fields, we did not observe any significant differences in receptive field location or area across different SR subtypes (data not shown). We were, however, able to compare response metrics for drifting grating and natural scene stimuli. We first investigated neurons responsive to drifting gratings, comparing the OSI (DG OSI) metric measuring how narrowly tuned a neuron is for oriented drifting gratings (1 = narrowly tuned, 0 = broadly tuned; Materials and Methods). SR and non-SR neurons had similar OSI distributions (Fig. 5*B*); the only significantly different comparison was between the OSI of non-SR neurons and that of nasally selective SR neurons, suggesting that nasally selective SR neurons may to a lesser extent discriminate the orientation of the DG stimulus. However, this difference may be caused in part by the small number of nasally selective SR neurons (*n* = 147) relative to other categories, and overall reflects negligible differences in orientation selectivity among SR and non-SR neurons. Next, we investigated neurons responsive to natural scenes (118 different natural images shown to mice every 250 ms), comparing the lifetime sparseness metric neuron capturing how selective a neuron is to the images shown (1 = responsive only to a single image, 0 = broadly responsive to all images; Vinje and Gallant, 2000; de Vries et al., 2020). If SR neurons had significantly lower lifetime sparseness values compared with non-SR neurons, then this may suggest they are more broadly responsive to abrupt changes in visual stimulus (e.g., those induced by a rapid eye movement). The majority of both SR and non-SR neurons had high lifetime sparseness values, but the distribution for the nasally selective SR neurons was slightly and statistically significantly lower than the other distribution, suggesting that these neurons may respond more broadly to visual stimuli, though again this difference may be because of small sample size (*n* = 96 nasal SR neurons; Fig. 5*C*). All other distributions of non-SR neurons, temporally selective SR neurons, and non-DS SR neurons were not significantly different (*p* > 0.05, KS test), suggesting that these latter types of SR neurons exhibited similar responses to visual scenes compared with those of non-SR neurons.

We finally considered the possibility that SR neurons were responding to the saccade-induced translation of the visual field. Specifically, if saccade responses are driven by visual field motion, we would expect SR neurons to respond preferentially to visual motion along the horizontal axis, as this is the primary axis of saccadic eye movements. Furthermore, we would expect DS SR neurons to respond to visual motion in the opposite direction as their preferred saccade direction, since this is the direction of visual field motion when the eye moves in the preferred saccade direction. To identify whether this was the case, we analyzed drifting grating tuning metrics, first comparing the DSI (DG DSI), a metric comparing the response of the neuron to its preferred and nonpreferred directions of motion for the drifting gratings stimulus (Fig. 5*D*; Materials and Methods). We found no significant difference between the distributions of SR neurons and non-SR neurons, suggesting that SR neurons are not differentially selective to specific directions of motion (two-sample KS test, p>0.05, with Bonferroni correction). Finally, we compared the preferred drifting grating direction of motion (the direction that maximizes the neural response) across neurons (de Vries et al., 2020). Again, we found no significant differences in these distributions between SR and non-SR neurons, suggesting that SR neurons do not differentially prefer motion along the horizontal axis (Fig. 5*E*). These two results taken together indicate there are no notable differences in visual motion responses of SR neurons relative to those of non-SR neurons, suggesting that the saccade-induced visual field motion does not primarily drive the responses of SR neurons.

### Spontaneous SR neurons detected from saccade responses during spontaneous stimulus similarly exhibit no distinct visual response properties

As SR neurons examined in the sections above were identified using saccades made while viewing different visual stimuli, we considered the possibility that the dynamic visual stimulus patterns could influence both SR identification and whether SR neurons had distinct visual response properties. To control for this, we identified “spontaneous SR neurons” by applying the same SR neuron detection procedure to neural activity and saccades during only the spontaneous stimulus (mean luminance gray screen; Materials and Methods). Though there was considerable variability in the quantity of spontaneous saccades across sessions (Fig. 1*G*), on average ∼10% of the saccades were made during spontaneous stimuli, dramatically reducing the statistical power (mean ± SD, *n* = 25 ± 30 saccades during spontaneous stimuli; median = 13 saccades). Spontaneous SR neurons occurred at an overall frequency of 5%, slightly lower than that of SR neurons (9%), but we still observe the same trends of higher SR frequencies in inhibitory populations and deeper-layer excitatory cell types. Likewise, the spontaneous SR neurons show the same strong bias for temporal saccades, with a positively skewed saccade response DSI (compare Extended Data Fig. 5-1*A*,*B*, Fig. 2*F*,*G*).

We do observe some differences in the distribution of spontaneous SR neurons across functional clusters. Whereas the frequency of SR was relatively uniform across functional clusters using all saccades, we find a decrease in the fraction of spontaneous SR neurons in the None cluster while the other clusters remain mostly the same (compare Extended Data Fig. 5-1*C*, Fig. 5*A*). If a visual feature or pattern were contributing to the SR designation, we would expect that identifying SR neurons using only the spontaneous epoch would decrease the number of SR neurons in the visually responsive clusters more than the None cluster. That is because neurons that have putatively visually driven saccade responses would be clustered in the visually responsive clusters but crucially should not be selected using the spontaneous SR method, resulting in a net SR frequency decrease in these visual clusters. Instead, we see only small decreases in the fraction of SR neurons in the visually responsive clusters but the largest decrease in the None cluster. This further supports the likelihood that visual features are not driving the saccade responses of these neurons. Indeed, comparing the tuning properties and visual preferences measured here across spontaneous SR subtypes, we again see that there are no distribution differences in OSI, DSI, lifetime sparseness, and preferred direction, consistent with what we observed when using all saccades (compare Extended Data Fig. 5-1*D*,*F*,*G*, Fig. 5*B*,*D*,*E*).

In summary, visual response properties—to the extent that can be measured using the stimuli in this dataset—do not differ strongly between SR neurons and the overall neural population, suggesting that saccade responses are not primarily visually driven.

## Discussion

In this study, we examined saccadic eye movements and visual cortical neural responses in head-fixed mice. These mice tended to make spontaneous saccades, with a frequency independent of the visual stimulus, occurring frequently in successive bursts, and preferentially along the horizontal direction. SR neurons were found across all cortical areas, layers, and transgenically defined cell types that were sampled in this dataset, though with differing prevalence across these dimensions. Previous work has found the dynamics of SR neurons are similar in both freely moving and head-fixed mice (Miura and Scanziani, 2022), and this suggests that the distributions we have found of SR neurons across cell type, cortical layer, and visual area should generalize to freely moving mice. Overall, our analysis reveals novel insights about the distribution of SR neurons and the lack of distinct visual response properties of these neurons across the visual cortex.

### Neural saccade response distributions across transgenic line, cortical layer, and visual area

SR neurons were found in all visual areas and all the of the cell types that were surveyed in this dataset; however, there were notable differences in the prevalence of SR neurons. First, SR neurons occurred more frequently in dorsal visual areas AL/PM/AM. This is consistent with egocentric representations and spatial processing in dorsal visual areas, but future work examining saccade responses in more ventral visual areas is needed to determine whether there is a clear difference in the rates of saccade-responsive neurons in these two pathways. Second, inhibitory neural cell types (Sst;Ai148, Vip;Ai148, Pvalb;Ai162) exhibited consistently high SR rates across visual areas (Taniguchi et al., 2011). Past work has shown that the inhibitory microcircuit mediates both bottom-up and top-down effects on cortical representation (Zhang et al., 2014; Dipoppa et al., 2018), and future work is needed to fully understand how these inhibitory interneurons integrate saccade signals with other sensory and behavioral signals.

Finally, there was a bias for SR neurons to occur more frequently in deeper cortical layers rather than supragranular layers, consistent with findings in a recent electrophysiological study (Miura and Scanziani, 2022). Although aggregating all visual areas revealed many layer four excitatory cell types that responded to saccades (Fig. 4*E*), analysis in V1 specifically revealed lower SR rates in layer 4 (with the exception of Slc17;Ai93) and a prevalent SR bias toward cell types in layers 5 and 6 (Extended Data Fig. 4-1). These findings are consistent with the anatomic organization of inputs from SC to LP (Zhou et al., 2017), and LP to visual cortex, where projections target layer 5 in V1 and layer 4 in higher visual areas (Herkenham, 1980). We also found a relatively high SR rate in Tlx3;Ai148 neurons in V1, which are corticocortical projecting layer 5 excitatory neurons that have been shown to both provide direct feedforward input to and receive feedback from higher visual areas and the SC (Gerfen et al., 2013; Kim et al., 2015). Such neurons may be important in relaying visual information to sensorimotor centers like the SC that is subsequently used to determine eye movements, but future work is needed to probe the exact details of this complex circuit (Gandhi and Katnani, 2011). This high rate of Tlx3;Ai148 neurons, in contrast to the corticofugal Fezf2;Ai148 layer 5 excitatory neurons (Guo et al., 2013), may implicate their importance in modulating levels of feedforward inputs and suggests that saccadic activity is differentially represented in these distinct pathways. Additionally, the Ntsr1;Ai148 transgenic line, labeling the majority of excitatory layer 6 neurons (Gong et al., 2007), had the highest SR rate of any excitatory neural cell type. Given that a distinct subpopulation of these Ntsr1;Ai148 neurons have apical dendrites extending to L1 (Olsen et al., 2012), the high SR rate we observed in this population is consistent with the notion that LP relays saccade signals to visual cortex through projections to superficial cortical layers (Roth et al., 2016; Miura and Scanziani, 2022). Moreover, given that these L6 neurons project both to superficial layers and to primary sensory thalamic nuclei (Olsen et al., 2012), they may also play a role in modulating visual representations through both intracortical and thalamocortical pathways.

Nonetheless, we emphasize the anatomic reality that many cortical neurons have layer-spanning dendrites, and it would be incorrect to attribute the saccade-modulated activity of the neuron to the layer of its soma. Thus, future research is needed to localize precisely where saccade signals enter cortex and leverage these insights to elucidate the role of bidirectional corticothalamic feedback in regulating sensory processing during saccades.

### Saccade-responsive neurons biased toward preferring temporal saccades

Saccade-responsive neurons with enhanced, direction-selective responses overwhelmingly favored saccades made in the temporal direction, and this bias was consistent across transgenic lines, cortical layers, and visual areas. Though other asymmetries in nasal and temporal saccade magnitudes (Meyer et al., 2020) and velocities (Maruta et al., 2006) have been previously documented, our finding of visual cortical neurons disproportionately favoring temporal saccades has not been documented and raises interesting questions about neural responses in mice. We found there was no clear preference for mice to make saccades in either the nasal or temporal direction; saccades in these two directions were made at approximately the same rates in individual sessions. Furthermore, our analysis of visual tuning curves suggests that these DS neurons do not differentially prefer horizontal visual motion in any specific direction, ruling out the explanation of this effect being because of a visual response bias toward horizontal motion.

One possible explanation, supported by our observation that the neural response was stronger with temporal saccades in all enhanced SR neurons (as opposed to only direction-selective neurons; Fig. 2*G*), involves differences in neural processing of monocular versus binocular vision. Mice have a central 40° binocular field of view, but the majority of their visual field is monocular, with retinal ganglion cells throughout the entire retina projecting to the contralateral hemisphere (Seabrook et al., 2017). Under this explanation, the bias toward preferring temporal saccades may be related to previously documented sensitivity to binocular disparity that is widespread across mouse visual cortex (La Chioma et al., 2019); this is ethologically relevant for discriminating stereoscopic depth, for example (Samonds et al., 2019). Another possibility is that because a fraction of nasal saccades in freely moving mice may be compensatory for optic flow in self-motion, it may be that the visual system has evolved to differentially enhance the response of saccade responses in the temporal direction.

### Retinotopic organization of saccade responses

Visual tuning properties, to the extent that could be investigated in this dataset, did not appear to differ across SR types. However, one open question that we were unable to resolve in this analysis is whether there is a retinotopic organization of saccade responses: is the prevalence of SR neurons and their bias to temporal saccades uniform across visual space, or is there an organization of these SR types across visual space that might suggest distinct ethologically relevant features? As the dataset analyzed in this study was collected to target neurons in the center of the gaze of the mouse, we were unable to examine this question (de Vries et al., 2020). While there is a small spread of receptive field locations in visual space, this spread is not large enough to address these questions rigorously. This remains an important and interesting open question.

### Visual versus motor responses

Saccade-responsive visual cortical neurons have previously been observed to respond to a mix of visual and motor saccade responses (Itokazu et al., 2018; Miura and Scanziani, 2022). If our SR neurons were consistently responsive to no visual stimuli (i.e., in the None class of visual response), then this may suggest the saccade response is largely non-visual, as the neurons exhibit little stimulus-driven visual response. On the other extreme, SR neurons with visual response properties deviating substantially from non-SR neurons, along with differences between SR-nasal and SR-temporal neurons (e.g., a different direction selectivity or preferred direction for drifting gratings), would implicate a largely visually driven saccade response.

However, we observed neither of these extremes (Fig. 5). First, SR neuron visual response properties overlapped heavily with those of non-SR neurons, indicating that SR neuron visual response properties are not substantially different from what we would expect by taking a random subset of the neuronal population. This suggests that the saccade responses of the neurons we identified are not uniquely visually driven, and conversely, neurons responsive to a particular visual stimulus are not differentially likely to respond to saccades. Second, SR neurons in our dataset tended to exhibit time-locked saccade responses during all visual stimuli—including the spontaneous stimulus (mean luminance gray screen), during which there would be a negligible visual response induced by a change in the visual receptive field. These observations indicate that the saccade responses here are not visually driven, but instead likely reflect motor signals being propagated through the cortex. It is worth reiterating, however, that there may be visual features to which SR subtypes respond differentially that are not used in this stimulus set (e.g., very fast motion speeds). This remains a possibility that we cannot confirm or rule out using these data.

We observed some minor heterogeneity, however, in the magnitude of saccade-responses observed during different visual stimuli, particularly in SR responses to saccades made during the spontaneous stimulus and those made during naturalistic stimuli (Fig. 3; natural movies and natural scenes). These latter stimuli have richer and more dynamic spatial statistics than other stimuli, driving greater overall cortical activity in both excitatory and inhibitory populations (de Vries et al., 2020). This suggests that visual signals do modulate neural saccade responses and supports a model of linear integration of these signals as proposed in Miura and Scanziani (2022).

## Synthesis

Reviewing Editor: Niko Busch, Westfalische Wilhelms-Universitat Munster Decisions are customarily a result of the Reviewing Editor and the peer reviewers coming together and discussing their recommendations until a consensus is reached. When revisions are invited, a fact-based synthesis statement explaining their decision and outlining what is needed to prepare a revision will be listed below. The following reviewer(s) agreed to reveal their identity: NONE.

# Synthesis

Both reviews are largely positive and highlight the merit of your study. However, the reviewers have a few requests for further clarifications, and I encourage you to carefully address each of the reviewers’ points. Specifically, several comments concerning the definition of saccade-related neurons could be addressed by additional or modified analyses of the existing data (see review #2).

# Reviewer 1

In this well-written manuscript, the authors take advantage of the large neural and behavioral dataset from the Allen Brain Observatory to investigate saccade responses in visual cortex of head-fixed mice. The manuscript goes through a series of careful analyses to describe the distribution of saccade-responsive neurons across cortical areas, layers and cell types. While it is difficult from the dataset to conclusively determine how much of the responses are visual vs motor, the authors compared the visual response properties of saccade-responsive and non-responsive neurons, which allowed them to infer that saccade-responses are not simply visually-driven. As the authors acknowledge, it has been previously shown that such saccade-responsive neurons exist in visual cortex, both in primates and rodents. This study is largely in agreement with the prior literature but expands the granularity of our knowledge on neuronal responses to saccades in visual cortex.

The results are detailed, clearly presented and merit publication in eNeuro. Nonetheless, I have some specific comments below, mostly minor, that I believe need to be addressed prior to publication.

L34: While it is true that the presence of neurons in mouse V1 that “modulate their responses to these saccadic eye movements” has been established, the specific citation Itokazu et al. (2018) concluded that there were very few such neurons in V1 (“In contrast, the V1, ..., showed little motor activity”). This may be explained by the fact that Itokazu et al analyzed the activity of L2/3 neurons, which the authors show in this manuscript have the smallest proportion of saccade-responsive neurons (L390). Other references, such as Roth et al. (2016) and Miura and Scanziani (2022) may be more appropriate here.

Can the authors explain in Methods how they converted the ellipse locations, which I assume are pixel locations, to degrees? Pupil size significantly alters the distance from the center of the eye to the plane that contains the pupil; this results in very different true angles for different pupil sizes even when the center positions appear identical on the video frame (see Stahl JS, Ann N.Y.Acad.Sci. 2002). What models were used to approximate the relationship between the pupil size and the angle?

Related to above, on L128, the authors state that the absolute eye speed was calculated with Pythagorean theorem using the speed calculated for azimuth and elevation. However, the eye moves as a sphere, and thus, the arc length, or the central angle between two points on a sphere, must rather be calculated using the spherical law of cosines.

L196: How was the p-value corrected for multiple comparisons? Given the large number of neurons, methods such as Bonferroni may be too conservative.

L287: I understand that this test (Wilcoxon signed-rank) was performed as a comparison to previous literature, and the authors proceed to perform a much more stringent test next. Nonetheless, the relatively large number of neurons compared to previous studies and the use of a set threshold p=0.05 would inevitably lead to a large number of false positives with the simple signed-rank test. Could the authors provide an estimate for the false discovery rate when using this method?

My understanding is that, to identify saccade-responsive neurons, all saccades were considered regardless of the visual stimulus (except for those closely preceded by another). Does this mean that the saccade conditions for each neuron were not identical? While the authors show that the relative frequency of saccades on average is not affected by visual stimuli shown, I would assume that the relative number of saccades made on different stimuli varied depending on the animal and the session. If the authors were to subsample saccades such that the relative number of saccades on different visual stimuli were matched across neurons, how would this affect the results, for instance in terms of the number of SR neurons? Alternatively, would it be better to define SR neurons based on a single visual stimulus condition, such as the gray screen?

Related to discussion L547, have the authors investigated the saccade response properties (i.e., nasal or temporal preference) in relation to the receptive field locations? Is the bias towards temporal saccades observed uniformly across the retinotopic map? Is there less temporal bias in neurons located in the binocular zone? Such an analysis, if dataset allows, would expand the manuscript and may provide interesting insights into the relationship between saccade response and binocular disparity.

# Reviewer 2

The article titled, “Saccade-responsive visual cortical neurons do not exhibit distinct visual response properties” presents results from new analyses of data previously published and available from the Allen Institute database. I think they are addressing interesting questions and the data are important to understanding the relationships between eye movements and visual processing. I have one major concern that tempers my enthusiasm though that mostly could be addressed by simplifying the presentation and analysis to focus on the most robust results or including additional analysis to rule out potential confounds.

My major concern is that visual responses following a saccade can be caused most obviously from a change in the relationship between the stimulus and the receptive field of a neuron. Saccades vary in size that are more than large enough to move stimulus features into and out of receptive fields. The saccades do not have to be even that big though. Because mice have mostly simple cell receptive fields, even movements large enough to move stimulus features into and out of subfields is enough to cause an increase or decrease in response. *This can easily be ruled out by only using the saccade data measured during spontaneous activity and comparing visual response properties between neurons with and without significant saccade driven activity.* The more difficult solution is to try to match receptive fields, saccade sizes, and stimuli. I doubt that is reasonable or even possible. This is partly because based on Fig. 1 in de Vries et al. 2020, eye movements were measured in the ipsilateral eye while stimuli were presented in the contralateral eye. Therefore, the actual saccade size is unknown. As this submission and previous studies have noted, nasal saccades are larger than temporal saccades, which means that although mouse saccades are mostly “conjugate”, the saccades actually differ in size between the eyes and those differences have a somewhat broad and unpredictable distribution (other than the nasal bias). Indeed, Fig. 2 suggests that saccade-enhanced responses are observed more often for temporal saccades. That would coincide with the mostly larger nasal saccades happening in the contralateral eye that is viewing the stimuli. Does this bias also happen during spontaneous stimulation? Related to all of this, it might be better to compare receptive field sizes and F1/F0 ratios between neurons with and without saccade-related activity. These visual response properties would probably have a stronger relationship to neurons enhanced or suppressed by saccades compared to OSI, DSI, and lifetime sparseness.

Minor comments:

173-174: I believe it is supposed to be significant saccade and not significant visual?

269-289: Are these animals trained at all? Habituated to the setup/stimuli/etc.? These factors influence saccade rates so it is worth mentioning their behavioral state/experience.
