# Supplementary figures and images for: Saccade-Responsive Visual Cortical Neurons Do Not Exhibit Distinct Visual Response Properties
Source: eNeuro. 2023 Sep 13;10(9):ENEURO.0051-23.2023. doi: 10.1523/ENEURO.0051-23.2023 (PMC10506534; doi:10.1523/ENEURO.0051-23.2023)

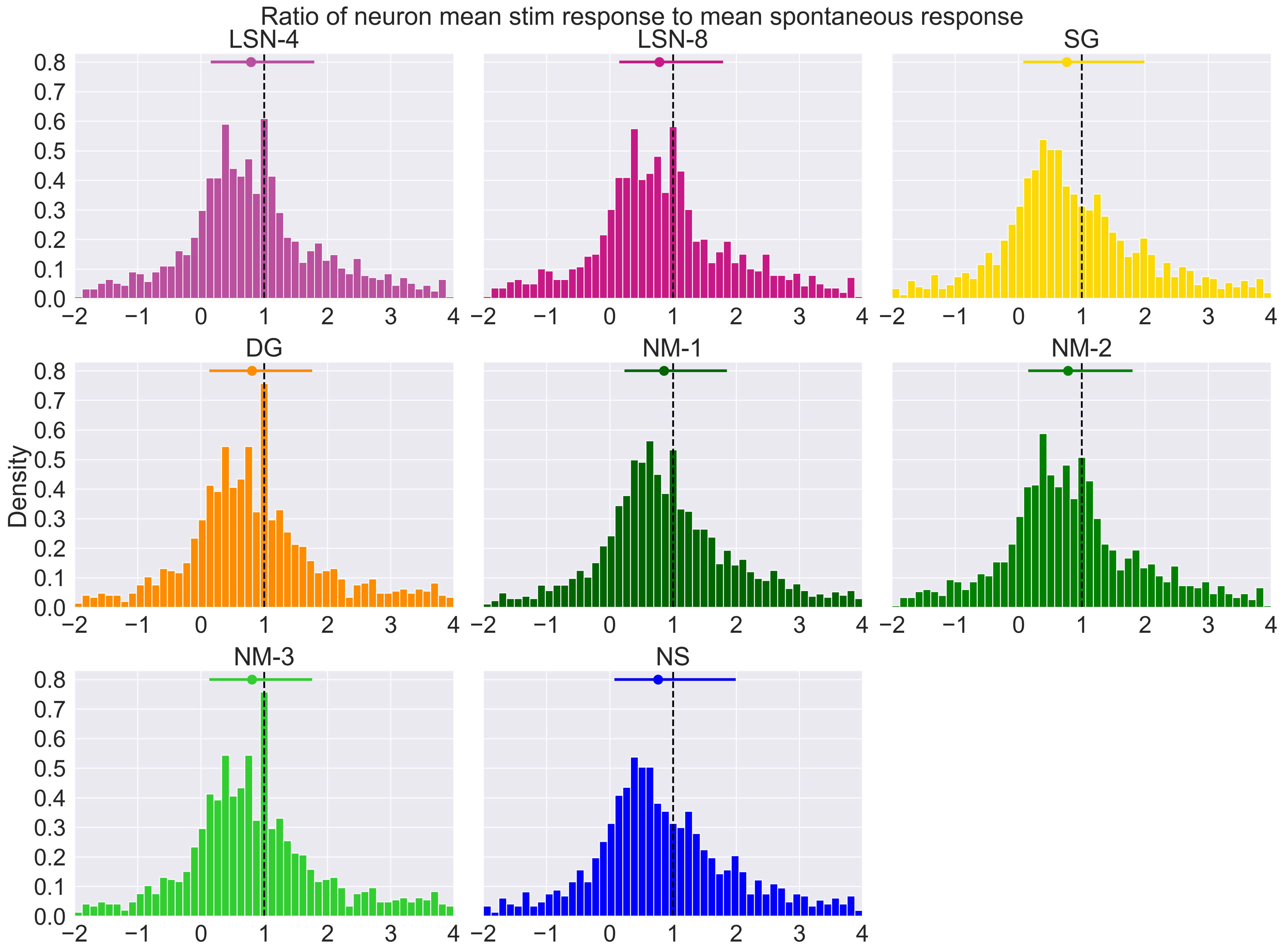

Supplement: Figure 3-1 — Ratio of the mean saccade response during each stimulus to the mean response during gray screen. Dashed vertical line at x = 1 indicates an equal mean response. Top bar and dot represent middle 50% and median, respectively. Download Figure 3-1, TIF file. [file enu-eN-NWR-0051-23-s02.tif]

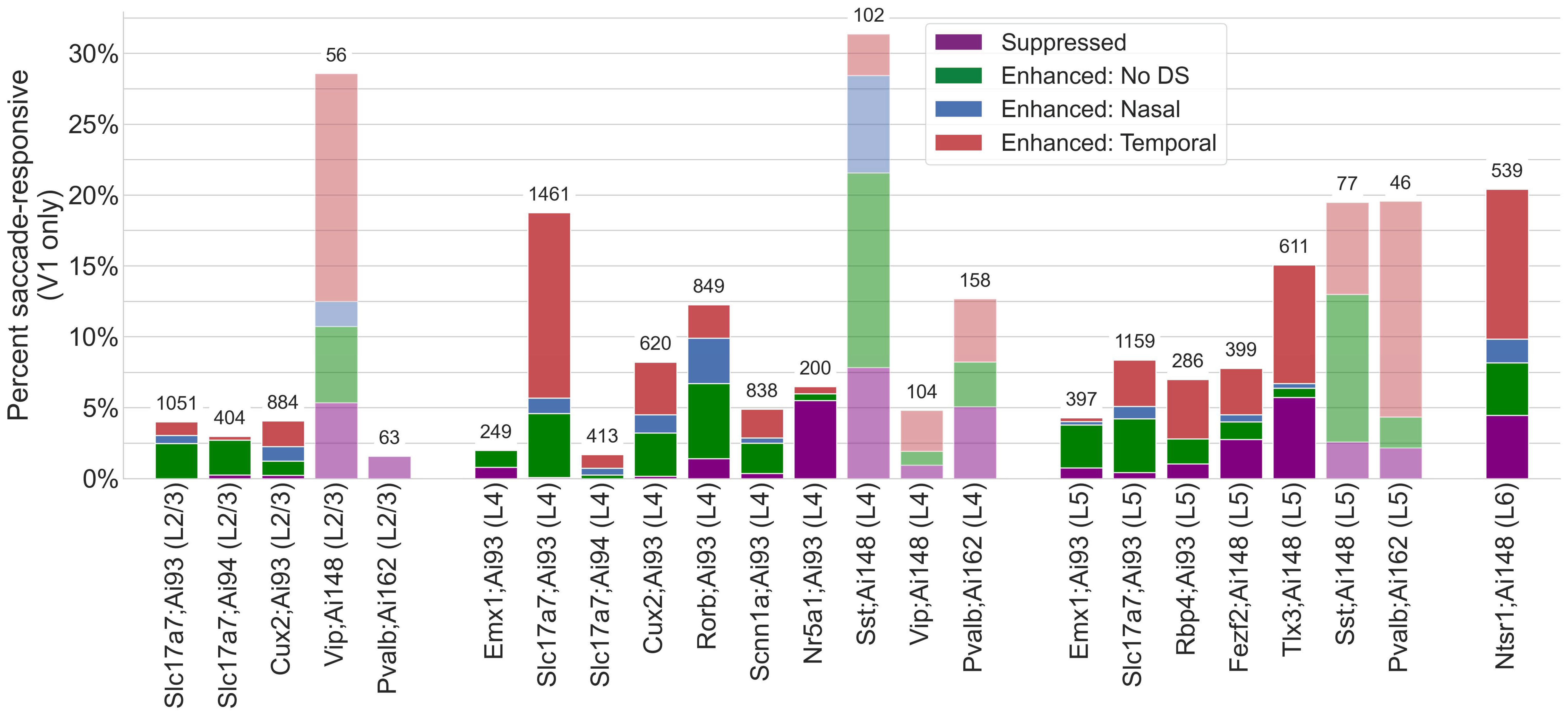

Supplement: Figure 4-1 — Percentage of saccade-responsive neurons in V1. Percentage of SR neurons by transgenic line and cortical layer, across only V1. Low-opacity bars indicate inhibitory neurons, and the numbers above bars indicate the total number of neurons imaged. See Extended Data Figure 5-1 for an analogous figure where SR neurons are detected using saccades during the spontaneous visual stimulus (Materials and Methods). Download Figure 4-1, TIF file. [file enu-eN-NWR-0051-23-s03.tif]

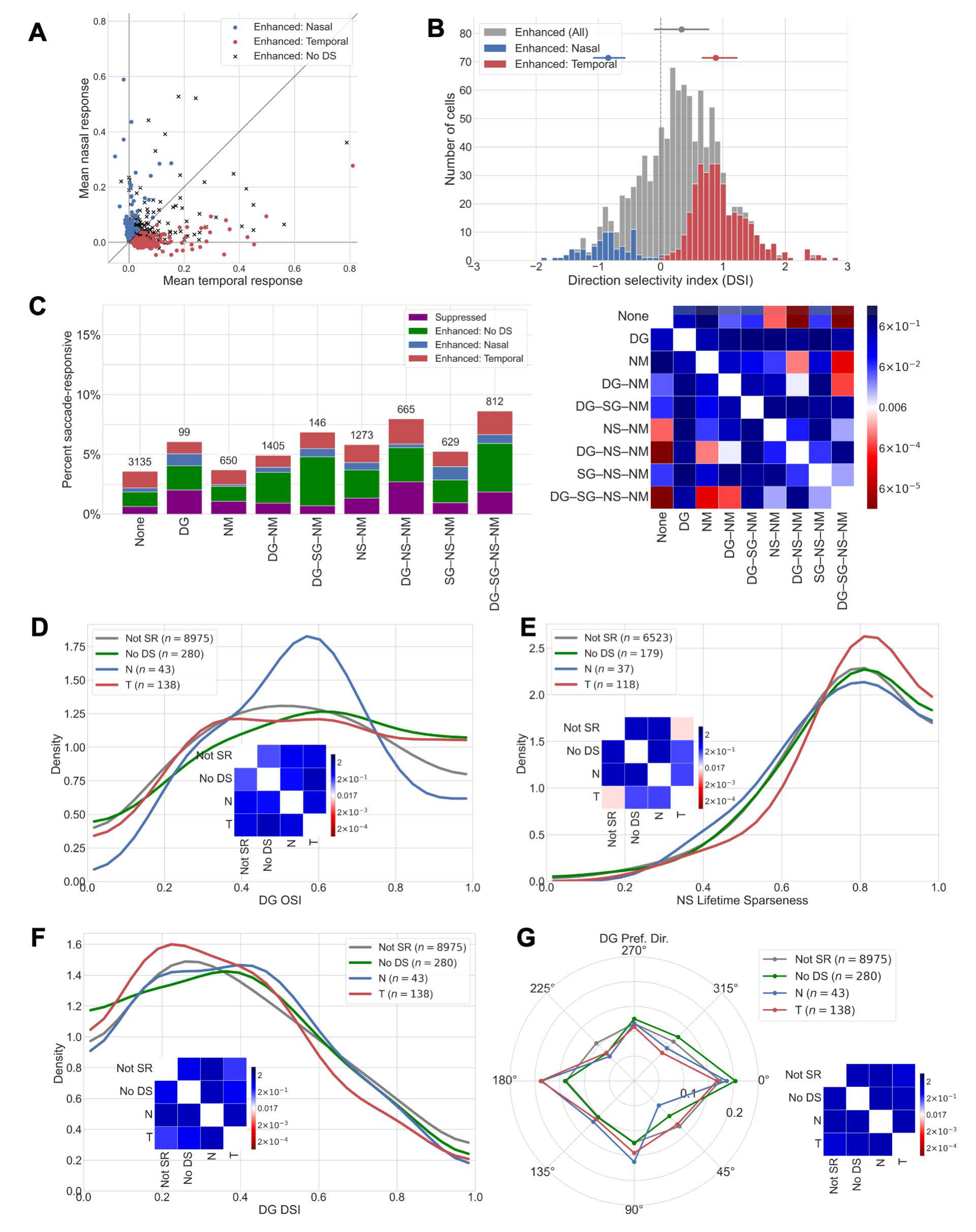

Supplement: Figure 5-1 — Spontaneous saccade-responsive neurons have similar visual responses to non-saccade-responsive neurons. Subplots A–E are analogous to those in Figure 5A–E, except that SR neurons in this figure are detected using saccades during spontaneous stimulus (Materials and Methods). A, Left, Percentage of SR neurons by visual response class, indicating which set of visual stimuli elicit a response. The number above each bar is the total number of imaged neurons within the given cluster. Right, χ2 test across pairings of transgenic lines (contingency matrix containing the number of SR and non-SR neurons for each cluster; p = 0.05, Bonferroni corrected for multiple comparisons). B, Orientation selectivity index for neurons that respond to drifting gratings. Curve is smoothed using a Gaussian kernel. Inset: Heatmap shows KS test between different distributions (p = 0.05, Bonferroni corrected for multiple comparisons). C, Analogous plot to B, showing lifetime sparseness for neurons responsive to natural scenes. D, Analogous plot to B, showing a direction-selectivity index plot for neurons responsive to drifting gratings. E, Distribution of preferred direction for neurons responsive to drifting gratings. F, Compare with Figure 2F. Scatter plot of saccade-responsive enhanced neurons showing their mean temporal saccade response (x-axis) and mean nasal saccade response (y-axis). Blue dot indicates that the neuron prefers nasal saccades, red dot indicates that neuron prefers temporal saccades, and black “x” indicates that the neuron is not direction selective. G, Compare with Figure 2G. Direction selectivity index histogram for saccade-responsive enhanced neurons. Gray, All saccade-responsive enhanced neurons (n = 1,026); blue, neurons that prefer nasal saccades (n = 81); red, neurons that prefer temporal saccades (n = 342). The bar is the middle 50% (all: –0.11, 0.78; nasal, –1.08, -0.57; temporal, 0.66, 1.23); dot is the median (all, 0.34; nasal, –0.84; temporal, 0.89). Download Fi [file enu-eN-NWR-0051-23-s04.tif]
